# Effects of an Electric Field on White Sharks: *In Situ* Testing of an Electric Deterrent

**DOI:** 10.1371/journal.pone.0062730

**Published:** 2013-05-02

**Authors:** Charlie Huveneers, Paul J. Rogers, Jayson M. Semmens, Crystal Beckmann, Alison A. Kock, Brad Page, Simon D. Goldsworthy

**Affiliations:** 1 Threatened, Endangered and Protected Species Sub Program, South Australian Research and Development Institute – Aquatic Sciences, Adelaide, South Australia, Australia; 2 School of Biological Sciences, Flinders University, Adelaide, South Australia, Australia; 3 Fisheries, Aquaculture and Coasts Centre, Institute of Marine and Antarctic Studies, University of Tasmania, Hobart, Tasmania, Australia; 4 Department of Zoology, University of Cape Town, Cape Town, Western Cape, South Africa; 5 Shark Spotters, Cape Town, Western Cape, South Africa; Universität Bielefeld, Germany

## Abstract

Elasmobranchs can detect minute electromagnetic fields, <1 nVcm^–1^, using their ampullae of Lorenzini. Behavioural responses to electric fields have been investigated in various species, sometimes with the aim to develop shark deterrents to improve human safety. The present study tested the effects of the Shark Shield Freedom7™ electric deterrent on (1) the behaviour of 18 white sharks (*Carcharodon carcharias*) near a static bait, and (2) the rates of attacks on a towed seal decoy. In the first experiment, 116 trials using a static bait were performed at the Neptune Islands, South Australia. The proportion of baits taken during static bait trials was not affected by the electric field. The electric field, however, increased the time it took them to consume the bait, the number of interactions per approach, and decreased the proportion of interactions within two metres of the field source. The effect of the electric field was not uniform across all sharks. In the second experiment, 189 tows using a seal decoy were conducted near Seal Island, South Africa. No breaches and only two surface interactions were observed during the tows when the electric field was activated, compared with 16 breaches and 27 surface interactions without the electric field. The present study suggests that the behavioural response of white sharks and the level of risk reduction resulting from the electric field is contextually specific, and depends on the motivational state of sharks.

## Introduction

Electro-reception is the ability to sense electrical stimuli, which is an ancient sensory capability that has been lost and re-evolved several times [1]. It is present in various vertebrates, including all elasmobranchs [2], most non-teleost fishes [3], [4], four orders of teleosts [5], caecilian and urodele amphibians [6], the platypus (*Ornithorhynchus anatinus*) [7], and the Guiana dolphin (*Sotalia guianensis*) [8]. In elasmobranchs, electromagnetic fields are detected by the ampullae of Lorenzini located in the head of sharks and in the head and pectoral fins of skates and rays [2]. Each ampulla functions as an independent receptor that measures the electric potential difference between the ampullary pore opening and the body interior [9]. Although the role of these gel-filled pores is not completely clear, several functions of the ampullary electrosense have been proposed, including detection of prey [10]–[14], predators [15], [16], and mates [17], social communication [16], [18], and magnetoreception/geonavigation [19]–[21]. Sensitivity to electric fields is comparable among elasmobranchs [13], [14], [22], yet the behavioural responses to electric fields can vary between species [22]. Elasmobranchs have shown behavioural responses to levels as low as <1nVcm^–1^ [22], [23].

Researchers have investigated whether the electroreceptive capabilities of sharks can be used to repel them from humans since the 1960s. Sharks have been demonstrated to be deterred when exposed to strong (3–7 Vm^–1^) localised electric or magnetic fields [24]–[28]. This led to the concept of using electrical fields to create repellents to reduce the probability of an attack [29]. Although shark attacks are rare, their impacts on humans can have serious and/or fatal consequences. Globally, the number of shark attacks between 1990–1999 and 2000–2010 has been increasing [30], [31]. This rise coincides with an increasing human population, more people visiting beaches, a rising popularity of the coastal lifestyle and marine activities, and increased accessibility of previously isolated coastal areas [30], [31]. Substantial efforts are being made to reduce the probability of shark attacks, with the behavioural response of sharks to electro-magnetic fields being seen as a promising means to deter sharks [28], [29].

**Figure 1:**
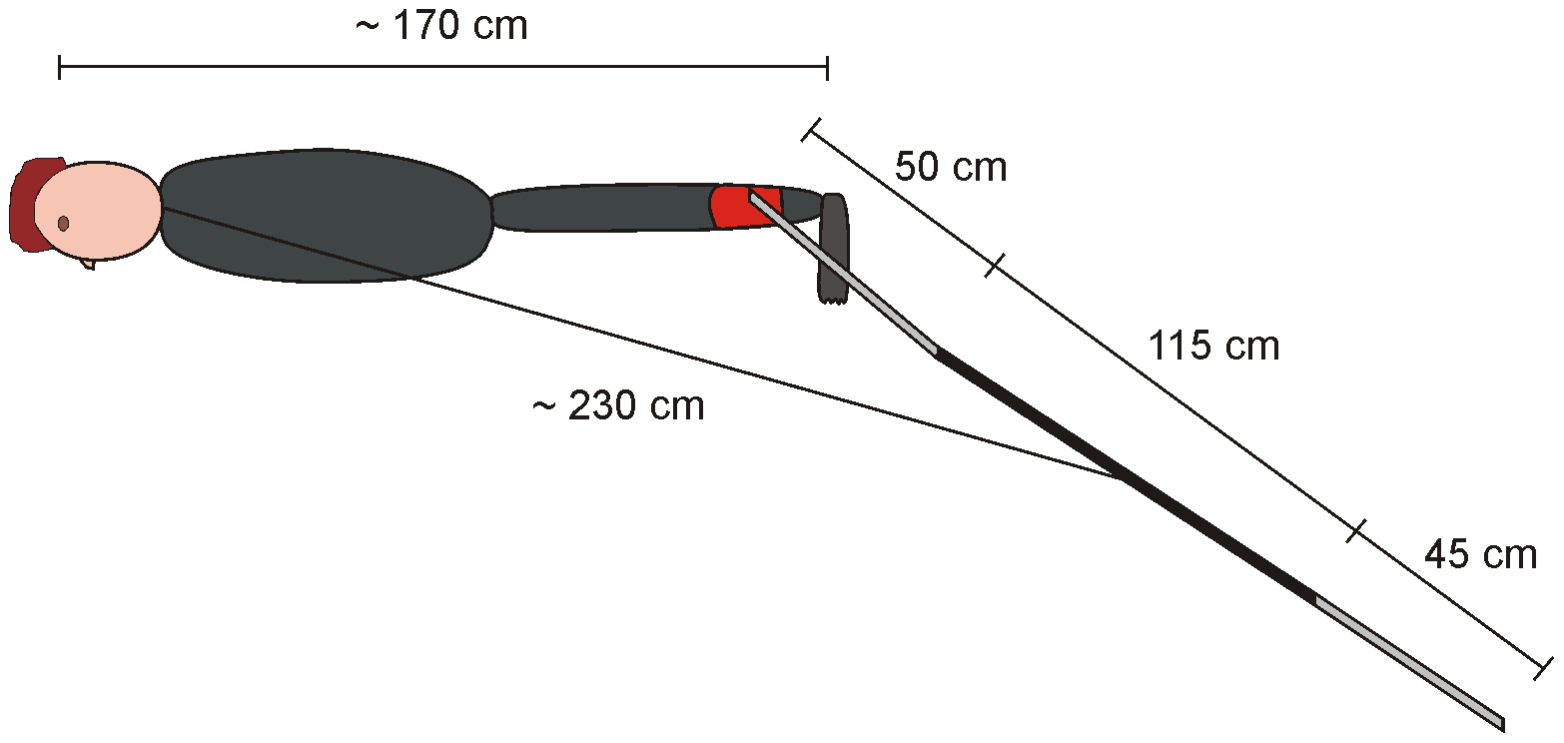
Schematic representation of a diver wearing a Shark Shield Freedom7™. The electrodes of the Shark Shield are represented in light grey. The electronic component that attaches to the ankle of the diver is represented in red.

While several studies have investigated the behavioural response of elasmobranchs to electric fields [22], [32], most were conducted under laboratory conditions. A field study that tested the efficiency of a personal electric deterrent on white sharks (*Carcharodon carcharias*) concluded that the probability of an attack was reduced from about 0.70 in power-off mode to about 0.08 in power-on mode [29]. Limited information about approach distance and number of approaches was presented, which would have allowed for a better understanding of the behavioural response of white sharks to electric deterrents. Additionally, the product tested (SharkPOD™) during this previous study is no longer available and has been replaced by the Shark Shield™ (Shark Shield Pty Ltd, Adelaide, Australia) product range. While the waveform and voltage difference between the electrodes produced by the Shark Shield™ is not different from that of the SharkPOD™, the electrode configuration differs between the two products. This results in differences in the maximum electric field produced and the distribution of the electric field relative to the body of the person using the device. The electrodes of the Shark Shield Freedom7™ trail behind the leg of the user (Fig. 1), while in the case of the SharkPOD™, one electrode is placed on the scuba tank with the other electrode on the ankle of the diver. As a result, the electric field source of the Shark Shield Freedom7™ is located behind the person wearing the device compared to being centred on the diver when wearing a SharkPOD™. Prior to this study, the Shark Shield Freedom7™ had not been independently and scientifically tested, and there remains a need to assess how different electrode configurations and locations of the electric field source may impact the efficiency of the electric deterrent.

The objective of this study was to assess the behavioural effects of the electric field produced by the Shark Shield Freedom7™ (hereafter referred to as the ‘electric field source (*EFS*)’) on white sharks (*Carcharodon carcharias*). This species was selected because it is responsible for the most unprovoked attacks and fatalities [30], [33]. White sharks demonstrate considerable plasticity in swimming patterns depending on their habitats and likely hunting strategies [34]. In response to this, we tested the effect of the electric field on white sharks in two different situations and locations: around a static bait at the Neptune Islands off South Australia, and breaching on a towed seal decoy at Seal Island, False Bay in South Africa.

## Methods

### Ethics Statement

This project was carried out under PIRSA Exemption 9902364, allowing us to engage in research activities within South Australia. The South African component of the project was conducted under the provisions of a permit from the Department of Environmental Affairs: Oceans and Coasts Branch (RES2010/74 and RES2011/40).

### Electric Field

The electric field was produced by the commercially available Shark Shield Freedom7™. This device produces exponentially decaying electrical pulses with an inter-pulse period of 0.6 s. Each pulse has a duration of about 1.2 ms and a peak amplitude of about 105 V (as measured *in vitro* in a tank filled with sea water). The pulses alternate in polarity. During normal use, the electrical circuitry and batteries of the device are worn on the ankle of the user (e.g. diver, snorkeler) with the centres of the two cylindrical electrodes (each of length ∼50 cm) 160 cm from each other (Fig. 1). The point halfway between the electrodes of the EFS is typically ∼230 cm away from the head of the user.

### Static Bait Experiments

#### Study site

**Figure 2:**
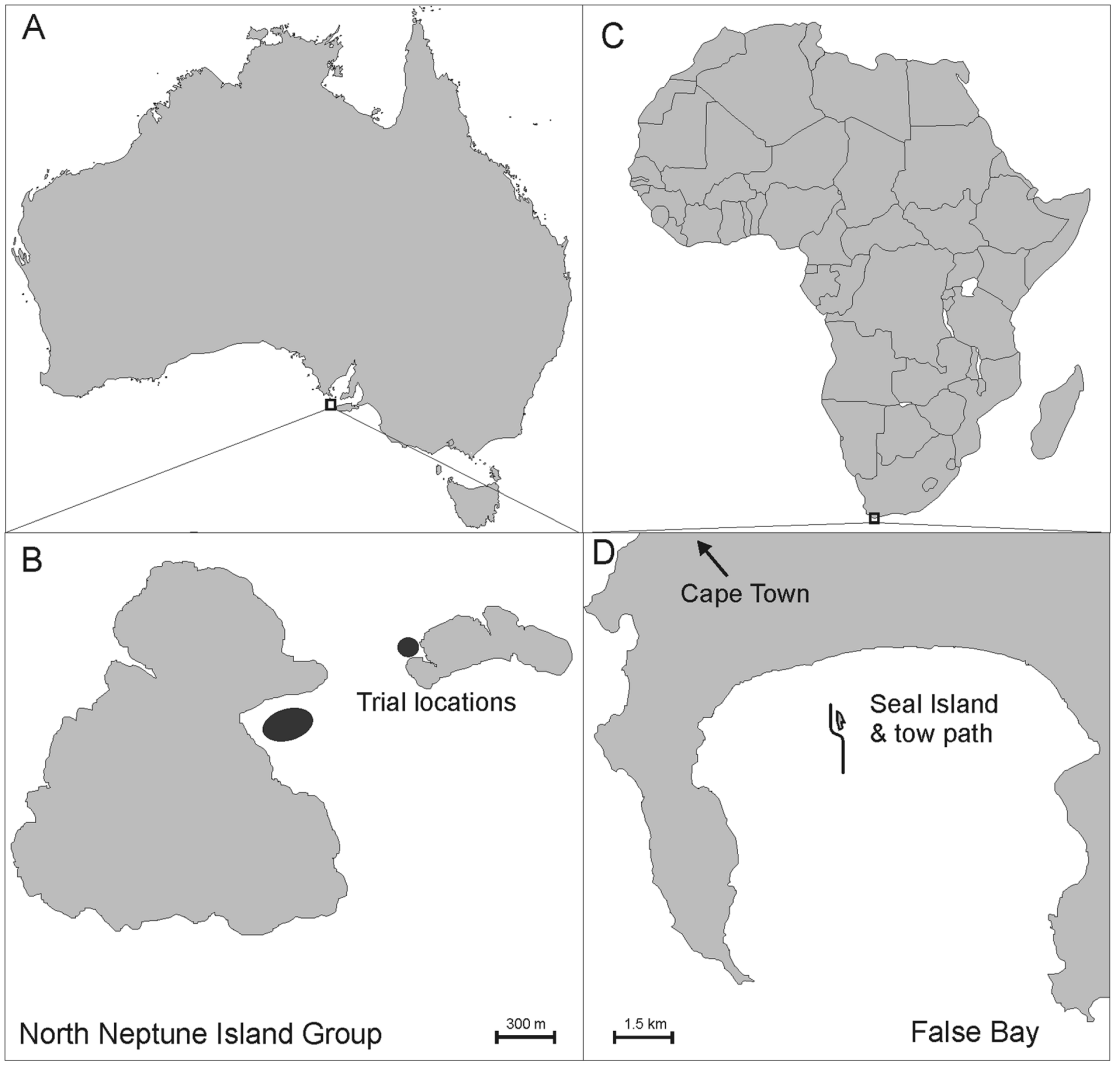
Location of (A) the North Neptune Island group, (B) where static bait experiments were undertaken, (C) False Bay, South Africa, and (D) Seal Island where dynamic tows were carried out.

The static bait experiments were undertaken at the North Neptune Island group (35°149 S; 136°049 E), located about 25 km south of Spencer Gulf. This site is considered the largest adult white shark aggregation in Australia [35] and provided the highest likelihood of obtaining sufficient interaction with the EFS. The Neptune Islands have been a commercial cage-diving site since the late 1970s, where white sharks regularly interact with static baits [36]. The experiments were carried out on three occasions: Trip 1∶11/10/2010–14/10/2010, Trip 2∶8/02/2011–10/02/2011, and Trip 3∶6/07/2011–7/07/2011 (Fig. 2A and B).

#### Experiments

White sharks were attracted to the stern of the anchored vessel using an odour corridor, which was established by continuously disbursing a mix of unrefined fish oil and minced southern bluefin tuna *Thunnus maccoyii* (SBT) flesh and blood, into the water. Sections of SBT were attached with short lengths of natural fibre to a float secured by a 15 m line. The SBT section was allowed to drift from the stern of the vessel to attract white sharks.

**Figure 3:**
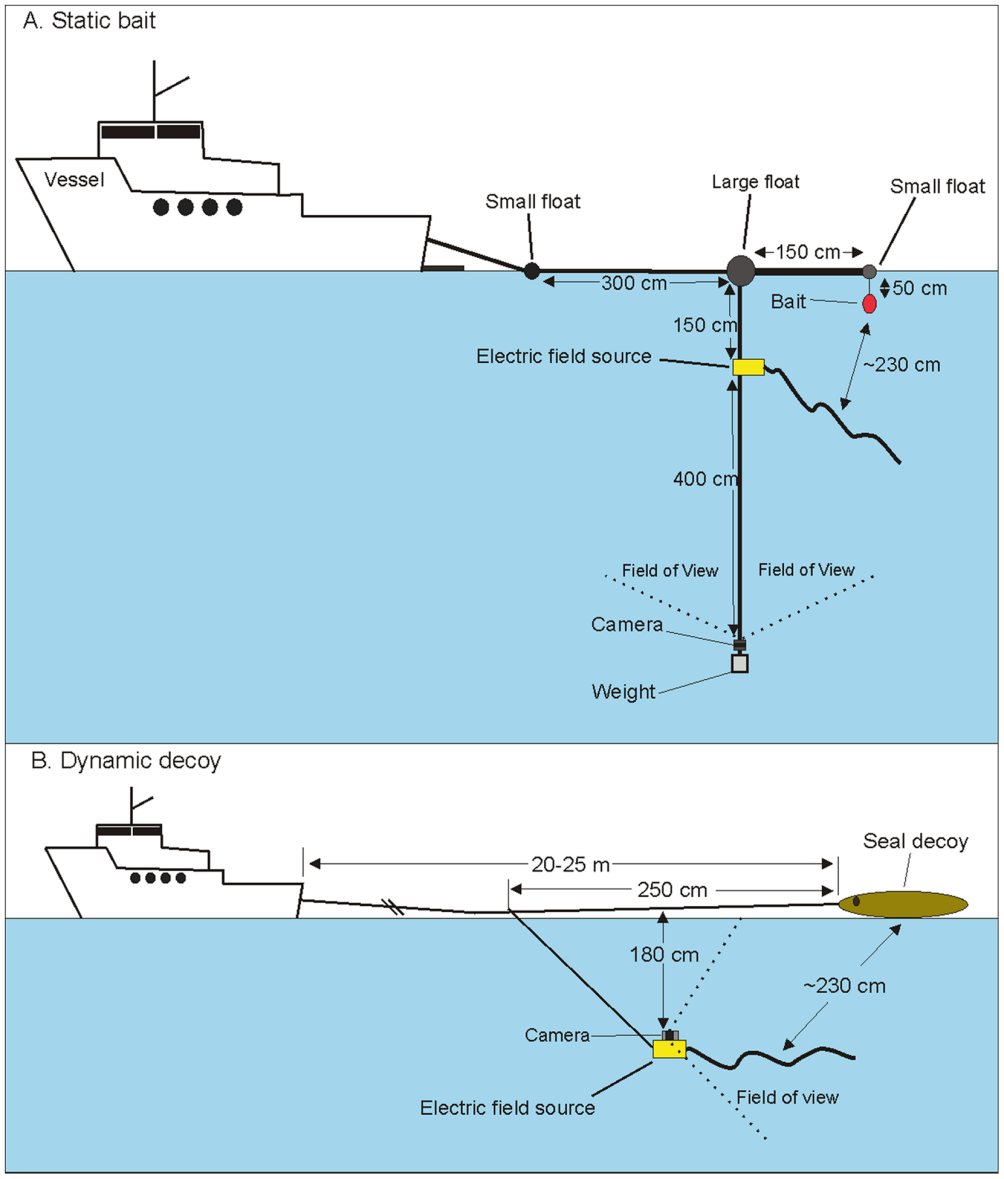
Schematic representation of (A) the experimental set-up used to test the effects of an electric field during static bait trials at North Neptune Island, and (B) the experimental set-up used with the towed seal decoy at Seal Island off South Africa. Reproduced and modified with permission from [52].

A total of 116 trials were completed, with 28, 64, and 24 trials during Trips 1, 2, and 3, respectively. Trials commenced after a white shark was sighted near the vessel at least twice within five minutes or when a shark showed consistent interest in the tethered bait. The tethered bait was removed and replaced with the experimental equipment, which was only deployed when the shark had left the proximity of the vessel and was no longer visible. Each trial consisted of the deployment of fresh SBT bait (*∼*6 kg). The head and tail sections of the SBT tuna were not used during the trials to keep the size and weight of the bait consistent. The bait was attached about 50 cm beneath a small foam float (15 cm diameter), which was separated from a large foam float (30 cm diameter) by a 150 cm long PVC pipe (Fig. 3). A 2-mm diameter plastic-coated wire of 550 cm in length was attached to the large foam float, with two ∼2 kg weights attached to its distal end. The EFS was attached to the wire 150 cm below the large foam float and a waterproof camera (GoPro™, California, USA) was attached at the end of the wire, 400 cm below the source of the electric field. The large foam float was connected to the stern of the anchored vessel using a rope, and was left to drift with the wind and tide. The distance of the equipment from the vessel varied between 5 and 15 m depending on the wind, swell, tide, and glare conditions, to ensure that observers on the vessel could identify sharks and record their behaviour accurately. Another small foam float (15 cm diameter) was attached 3 m from the large foam float on the line between the vessel and the large foam float to provide a known measurement and help with the estimation of shark total length and distance between the shark and the equipment (Fig. 3). The bait and small foam float were kept away from the ropes and wires to prevent sharks from biting them or becoming entangled. The minimum distance between the bait and the point halfway between the electrodes of the EFS was 100 cm with the maximum distance being ∼330 cm. The bait was mostly ∼230 cm from the EFS due to wind and current acting on the EFS and bait in a similar direction. The equipment was deployed to replicate the normal use of the EFS, where the point halfway between the electrodes of the EFS is ∼230 cm from the head of the user.

Each trial was observed by two people and lasted 15 minutes or until a shark took the bait. The status of the EFS (on or off) was randomised by a coin toss before each trial. The EFS was tested to ensure that electric impulses were being produced prior to and following each trial during which the EFS was switched on.

The following terminology was used to describe shark behaviour and to assess the effects of the electric field.

Approach – An *approach* was defined as when a shark was observed within 20 m of the static bait. In most situations, observers were not able to maintain visual contact with a shark when it was >20 m from the static bait.

Interaction – An *interaction* was defined as a directed swim towards the static bait. Each time a shark veered away from the bait and swam back towards the static bait, it was considered a new *interaction*. An *approach* was classified as having at least one *interaction*, but could have several interactions within an *approach* sequence. Video S1 provides an example of a white shark taking the bait preceded by one approach and one interaction. Video S2 provides an example of a white shark making one approach with six interactions before taking the bait.

Shark identity was recorded for each individual shark using natural markings and colouration [37]. Three physical features were used for shark identification: the trailing edge of the first dorsal fin [38], [39], the pigmentation of the lower caudal fin [37], and external markings or scars (e.g., fin damage, major scars).

Several variables were recorded and used to assess the behavioural response of sharks to the electric field produced:

1. Proportion of static baits consumed;
2. Time for the 1^st^ approach (hereafter referred to as ‘*approach time*’), defined as the time between deployment of the experimental gear and the first approach within 20 m from the bait;
3. Time taken to consume the bait (hereafter referred to as ‘*bait time*’), defined as the time between deployment of the experimental gear and when sharks consumed or bit the bait;
4. Number of approaches per trial;
5. Number of interactions per approach; and
6. For each interaction, the minimum distance between the snout of the shark, where its electro-sensory organs are located, and the EFS was recorded (hereafter referred to as *distance*). This distance was estimated through comparison of measured sections of the equipment: 300 cm between the main and small floats, 150 cm between the main float and float over the bait.

#### Coding of approaches and interactions

Digitally recorded video footage from each trial obtained from the underwater camera was reviewed, and independently and ‘blindly’ coded. Coding refers to recording the number of approaches and interactions, and estimating the minimum distance between the shark and the EFS during each interaction. The coder was termed ‘blind’ as they did not participate in the trials and had no prior knowledge of whether an electric field was being produced when coding videos of each trial. The observer data recorded during the trials were used to identify sharks responsible for each approach.

#### Data analysis

There were two potential analytical biases inherent in the data we collected: 1) temporal correlation (lack of temporal independence) due to the potential habituation of individual sharks or changes in their motivation through time, and 2) pseudo-replication due to instances where the same shark interacted with the bait within and across trials. Sharks may have become habituated to the electric field, or sharks that consumed the bait, may have become less likely to respond to the electric field due to the positive reinforcement provided by the bait.

Temporal correlation was tested by estimating the Pearson’s r and the significance of the correlation for each response variable across time. Replicates varied across response variables (e.g., a distance was estimated for each interaction, but one approach time was obtained per trial), therefore the time variable changed depending on the response variable being tested. Trial number was used for approach time, bait time, and number of approaches per trial, whereas approach number was used for the number of interactions per approach, and interaction number was used for the distance. The Pearson’s r were calculated independently for each trip because four months elapsed between the field trips, and different sharks were observed during each trip.

The proportion of baits taken by sharks was compared using the minlike two-sided Poisson exact test from the *exactci* R package (R statistical software, Ver. 2.13.1) [40]. The minlike two-sided method was chosen because it is generally more powerful than the central two-sided method [40].

Pseudo-replication was managed by testing the effects of the electric field for all other response variables using a Generalised Linear Mixed-Model (GLMM) with individual shark as the ‘random effect’ and whether the electric field was produced as the ‘fixed effect’. This could not be undertaken for the proportion of baits taken due to the small sample size. The error structure of GLMM corrects for non-independence of statistical units due to shared temporal structure, and permits the ‘random effects’ variance explained at different levels of clustering to be decomposed. The inclusion of individual shark as a random effect enabled the analysis to account for the lack of independence in behaviour within each identified shark. Each approach or interaction for which shark identification could not be determined was excluded from this analysis. The most appropriate statistical family and error distribution for each analysis was determined through the examination of the distribution of the response variable, a visual inspection of the residuals for the saturated models, and the Akaike Information Criteria value (measure of the relative goodness of fit of a statistical model) [41] when available (depending on the R function used between glmmPQL - library MASS, lmer - library lme4, and glmmML - library glmmML).

Finally, the effects of the electric field were tested by comparing the distributions of the minimum distance recorded for each interaction using a Kolmogorov-Smirnov (K-S) test [42] and by comparing the proportion of interactions within 2 m using the minlike two-sided Poisson exact test from the *exactci* R package.

### Dynamic Tow Experiments

#### Study site

The dynamic tow experiments were conducted off Seal Island, in False Bay, south of Cape Town, in the Western Cape region of South Africa (Fig. 2C and D). This site was chosen because it has a high recorded rate of predatory behaviour of white sharks on pinnipeds [43]. At this location, sharks are regularly observed to breach during natural predation events [44]. It was assumed that this predatory breaching behaviour would provide a good opportunity to test the effect of the electric field. Experimental decoy tows have been successfully used to study Cape fur seal predation risk when moving near Seal Island [45].

#### Tows of seal decoy

A fibreglass coated foam seal decoy was towed 20–25 m behind a vessel at speeds of 8–10 km.hr^–1^, which was based on travelling speeds of Cape fur seals leaving Seal Island [45]. To maximise the chance of eliciting a predatory response, the tow time and route were chosen based on the knowledge that predator-prey activity is spatio-temporally confined and predictable at Seal Island [43]. Tows were confined to the sunrise (low light) and mid-morning periods between 6∶30–10∶00 am. They were conducted in the area between 1 km south of Seal Island towards the Island and the southern tip of the island called the “launch pad” [43], to the West about 50–150 m from the Island, and the Northwest area of Seal Island (Fig. 2D). Tows were 1.7–2 km long and undertaken in both a North and South direction. A total of 189 tows were completed during 22 days of towing.

The EFS was affixed to a small black trolling paravane or underwater glider (175×75 mm) to ensure that the equipment glided through the water at a suitable angle to record shark approaches and interactions with the decoy. Two ∼900 g weights were attached to the paravane to bring the EFS to a water depth of *∼*180 cm and to prevent the EFS from streaming along the surface. An underwater camera (GoPro™) was fixed to the paravane to record interactions between sharks and the seal decoy, including those not visible from the surface (e.g. aborted breaches). The EFS, paravane, and camera were connected to the vessel via a length of 2-mm diameter wire to avoid the loss of the equipment in case of a physical interaction with a shark. The seal decoy was linked to the wire by a 1.2-mm diameter nylon fishing line of about 250 cm in length (Fig. 3). The equipment was configured so that the decoy was slightly behind the end tip of the EFS to reduce the potential for visual and/or physical distraction for a shark breaching. The distance between the seal-decoy and the point halfway between the electrodes of the EFS was *∼*230 cm. During each tow, the following data and observations were recorded: the start and end locations, duration of the tow, breaches and/or investigations, and other seal and shark activities. The status of the EFS (on or off) was randomised before each tow.

#### Selection of shark interactions and data coding

All digital recorded footage collected during the dynamic tow experiment was reviewed by CH. Once the interactions were identified from the video footage, they were isolated and clipped with Camtasia Studio 7.0 (TechSmith, Okemos, Michigan, USA) for further coding. Interactions during which shark behaviour could not be determined (e.g., due to low visibility, distance of the shark, and framing) were discarded to remove any ambiguous interactions. Each interaction was categorised as:

- A *breach*: interaction during which a shark leaps out of the water, with several sub-types described by Martin et al. [44] (Video S3 and S4);
- A *surface interaction*: interaction during which a shark does not leap out of the water but during which dorsal and/or caudal fins are visible above the water surface, such as during lateral roll, surface arc, direct or surface approach in Martin et al. [44] (Video S5); or
- An *underwater interaction*: interaction which is not visible from the surface (Video S6).

Seven scientists not present during the trials then categorised each underwater interaction into either an investigation or an aborted breach.

- An *investigation* was defined as any interaction during which a shark approached the decoy at a slow speed or at a vertical angle of less than 30° (Video S7). Speed was assessed using the time between shark appearance on the footage and when it got within 2 m of the seal decoy. Angle of approach was estimated by looking at the angle difference between the shark body and the water surface when 2 m away from the seal decoy.
- An *aborted breach* was defined as when a shark approached the decoy with speed and at an angle of more than 30° within 3 m, but did not complete the approach and did not breach the water surface (Video S8).

Each underwater interaction was also assessed as to whether it concluded with a sudden change of direction of more than 45° (potential response to the electric field, categorised as ‘yes’, ‘no’, or ‘unsure’) (Video S9). The level of confidence in the coding was recorded using a three-level confidence scale from one to three with one indicating a small amount of confidence in the coding assigned and three indicating a high level of confidence. Any coding data obtained with a confidence rating of one or with less than 70% agreement between coders, were excluded from the analysis to avoid including the interactions where coders were not confident in their interpretation or where coders disagreed,

#### Data analysis

The efficacy of the electric field in repelling white sharks from attacking a towed seal decoy was assessed by comparing the number of breaches, surface interactions, underwater interactions, and total number of interactions standardised by the number of replicates (i.e., the number of tows or number of videos) using the minlike two-sided Poisson exact test from the *exactci* R package [40]. A binomial distribution based on the probabilities of breaches and surface interactions occurring with the EFS activated was also used to estimate the probabilities of the observed number of breaches and surface interactions occurring when the EFS was off. The proportion of aborted breaches and investigations coded and the proportion of underwater interactions with a reaction to the electric field were tested using the same minlike two-sided Poisson exact test.

In the static and dynamic experiments, statistical tests were undertaken on data combining trips and years to ensure large sample sizes. For all statistical analyses, *p*<0.05 was considered statistically significant. Numbers in brackets represent mean ± standard error, unless stated otherwise.

## Results

### Static Bait Experiments

Forty-nine of the 116 trials were performed without an electric field and 67 with an applied electric field. A total of 314 approaches and 527 interactions by 18 different white sharks were observed. Identification of white sharks was not possible for 132 approaches (42%) and 179 interactions (34%). Sharks interacted with the bait in up to 27 trials (6.89±1.6, mean ± standard error). The number of approaches per identified shark ranged from 1 to 40 (10.11±2.5), while the number of interactions per identified shark ranged from 1 to 71 (19.33±4.9). During a single trial, the maximum number of approaches, interactions, and interactions per approach was 12, 29, and 18, respectively.

#### Temporal correlations

The behaviour of the sharks was consistent over time as little or no temporal correlation (r = 0–0.3) was found for any response variable (Table 1). A negative correlation was apparent during Trip 3 and suggested that the time it took sharks to approach the bait, to consume the bait and the number of approaches per trial decreased slightly as the trials were being undertaken. These correlations, however, were weak.

**Table 1:** Summary of Pearson’s r for each response variable of the static trials.

Response variable	Trip 1	Trip 2	Trip 3
Approach time	−0.153 (0.09)	−0.041 (0.11)	−**0.174 (0.04)**
Bait time	−0.182 (0.11)	−0.011 (0.45)	−**0.247 (0.02)**
Approaches per trial	0.013 (0.56)	0.001 (0.87)	−**0.300 (<0.01)**
Interactions perapproach	0.034 (0.25)	0.001 (0.90)	0.001 (0.96)
Distance	−0.114 (0.43)	0.037 (0.47)	−0.175 (0.13)

Approach time is the time between deployment of the experimental gear and the first approach within 20 m from the bait; bait time is the time between deployment of the experimental gear and when sharks consumed or bit the bait; distance is the minimum distance between a shark and the deterrent measured for each interaction. Numbers in brackets represent p-value; numbers in bold represent significant correlations (P<0.05). Reproduced and modified with permission from [52].

#### Effects of the electric field on the behaviour of white sharks

The bait was consumed on 91 out of 116 trials (78%), with the electric field not significantly affecting the likelihood of the bait being consumed (Poisson exact test: *p* = 1.00). Out of the 18 identified sharks that interacted with the bait, 14 (78%) consumed the bait, with 13 (72%) consuming the bait in the presence of the electric field. Six sharks consumed the bait on several occasions, with one shark consuming the bait a total of 23 times including 14 times when the electric field was being produced. Sharks responsible for consuming the baits could not be identified on 15 occasions (16%).

Out of the five response variables used to assess the effects of the electric field, the time it took to take the bait, number of interaction per approach, and the minimum distance between sharks and the EFS were significantly affected by the electric field (Table 2). Additionally, the random factor (individual sharks) was also significantly different for all parameters (Table 2).

**Table 2:** Summary of the Generalised Linear Mixed-Model results from the static trials.

Parameters analysed	DF	Shark ID (intercept)		EFS	
		*t*	*p*	*t*	*p*
Approach time	49	3.86	<0.001	−0.17	0.87
Bait time	61	5.54	<0.001	−2.58	0.01
Approach per trial	105	9.52	<0.001	0.87	0.38
Interaction per trial	163	2.42	0.02	3.66	<0.001
Distance	292	8.25	<0.001	2.6	0.01

Approach time is the time between deployment of the experimental gear and the first approach within 20 m from the bait; bait time is the time between deployment of the experimental gear and when sharks consumed or bit the bait; distance is the minimum distance between a shark and the deterrent measured for each interaction. DF represents degree of freedom; EFS is electric field source. Reproduced with permission from [52].

**Figure 4:**
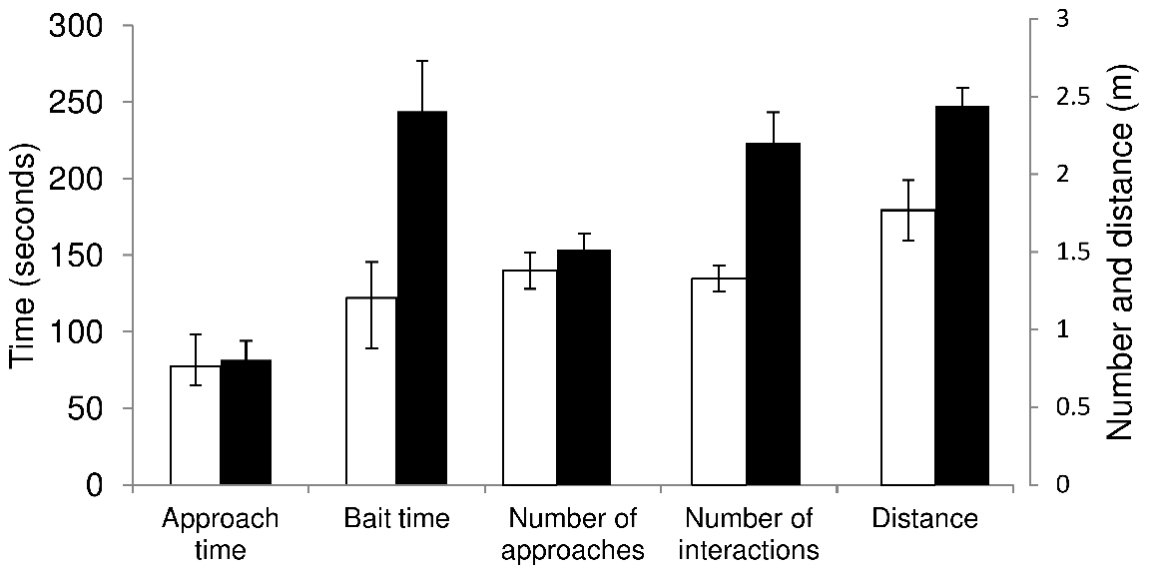
Effects of the electric field during the static bait trials. White bars represent trials with the electric field source (EFS) turned off; black bars represent trials with the activated EFS; standard error bars are shown.

Sharks first approached the bait within a short period (80±11 seconds). This was not affected by the electric field, with no significant difference in the time it took sharks to first be sighted whether the EFS was turned off (77±21 seconds) or on (82±12 seconds) (GLMM (Gamma, inverse): *t*_49_ = −0.17, *p* = 0.87; Table 3) (Fig. 4). Sharks took, on average, 197±23 seconds from the start of a trial to consume the bait. Although the electric field did not affect the time it took sharks to be first sighted, sharks took twice as long to take the bait when the EFS was turned on (244±32 seconds) than when it was turned off (122±24 seconds) (GLMM (Gamma, inverse): *t*_61_ = −2.58, *p* = 0.01; Table 3) (Fig. 4).

**Table 3:** Summary of the results obtained from the static bait experiment.

Parameters	OFF	ON	Total
N^o^ of trials	49	67	116
N^o^ of sharks	14	17	18
N^o^ of approaches	93	221	314
N^o^ of interactions	121	406	527
N^o^ of baits taken	38	53	91
Proportion of bait taken	77.6%	79.1%	78.4%
Mean approach time (sec)	112 (24)	69 (9)	87 (12)
Mean Bait time (sec)	163 (29)	233 (30)	204 (21)
Mean Approaches/trial	2.02 (0.20)	3.56 (0.37)	2.91 (0.24)
Mean Interactions/approach	1.30 (0.06)	1.84 (0.12)	1.67 (0.09)
Mean Distance	2.13 (0.20)	2.67 (0.10)	2.55 (0.09)

Numbers in brackets are standard errors; approach time is the time between deployment of the experimental gear and the first approach within 20 m from the bait; bait time is the time between deployment of the experimental gear and when sharks consumed or bit the bait; distance is the minimum distance between a shark and the deterrent measured for each interaction. This table summarises all data recorded, included for unidentified sharks.

There was no significant difference in the number of approaches per trial when the electric field was produced (GLMM (Poisson, identity): *t*_105_ = 0.87, *p* = 0.39) (Fig. 4). The number of interactions per approach, however, increased from 1.33±0.08 when the EFS was turned off to 2.20±0.20 when the EFS was turned on (GLMM (Poisson, log): *t*_163_ = 3.66, *p*<0.001; Table 3). This suggests that the sharks did not approach the bait more often when an electric field was produced, but interacted with the bait more often within each approach (Fig. 4).

**Figure 5:**
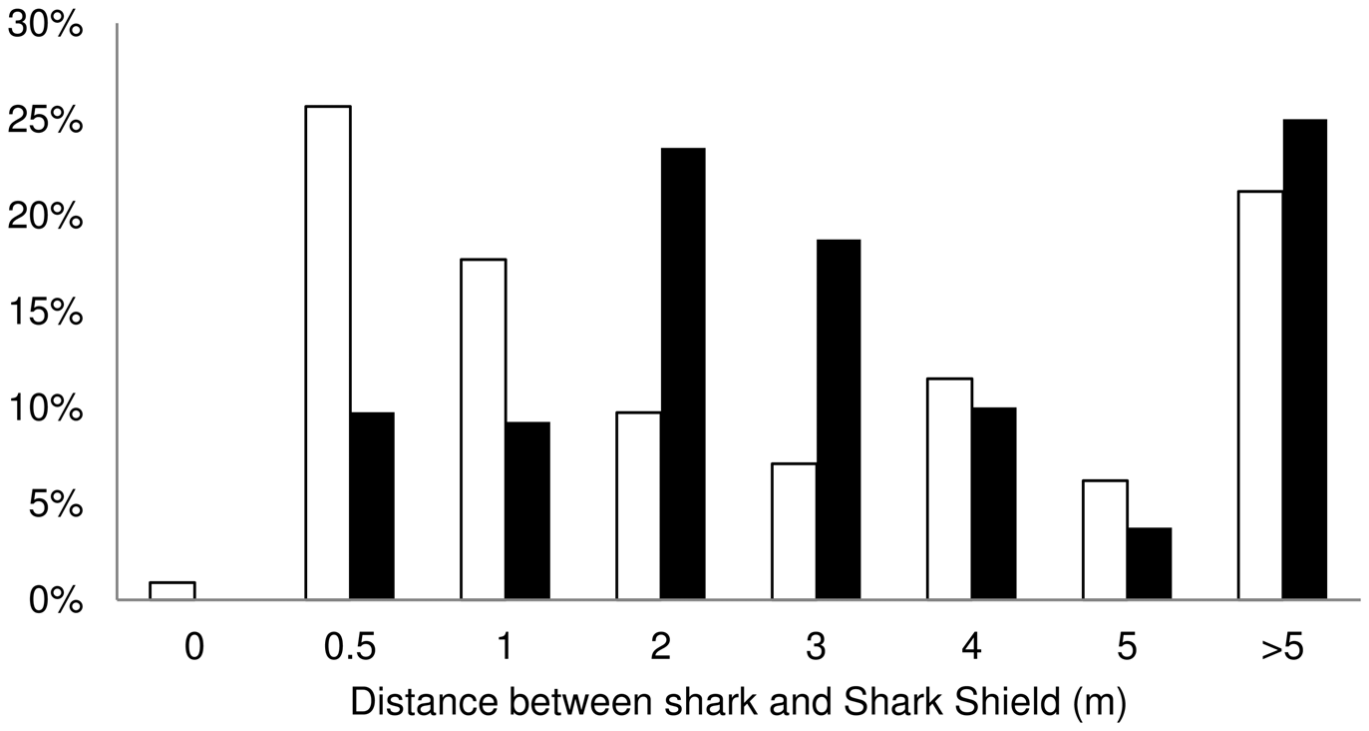
Histograms of the minimum distance between white sharks and the electric field source (EFS) when it was turned off (white) and on (black). Reproduced with permission from [52].

Although sharks were still able to consume the bait when the electric field was produced, it impacted the behaviour of the shark and significantly increased the mean minimum distance between the shark and the EFS from 1.77±0.20 to 2.44±0.11 m when activated (GLMM (Gamma, identity): *t*_292_ = 2.60, *p* = 0.01; Table 3) (Fig. 4). The distribution of the minimum distance between the sharks and the EFS also changed significantly (K-S test: *t* = 2.75; *p*<0.001), with less interactions within 2 m of the EFS when it was turned on (Poisson exact test: *p* = 0.0001) (Fig. 5).

### Dynamic Tow Experiments

**Figure 6:**
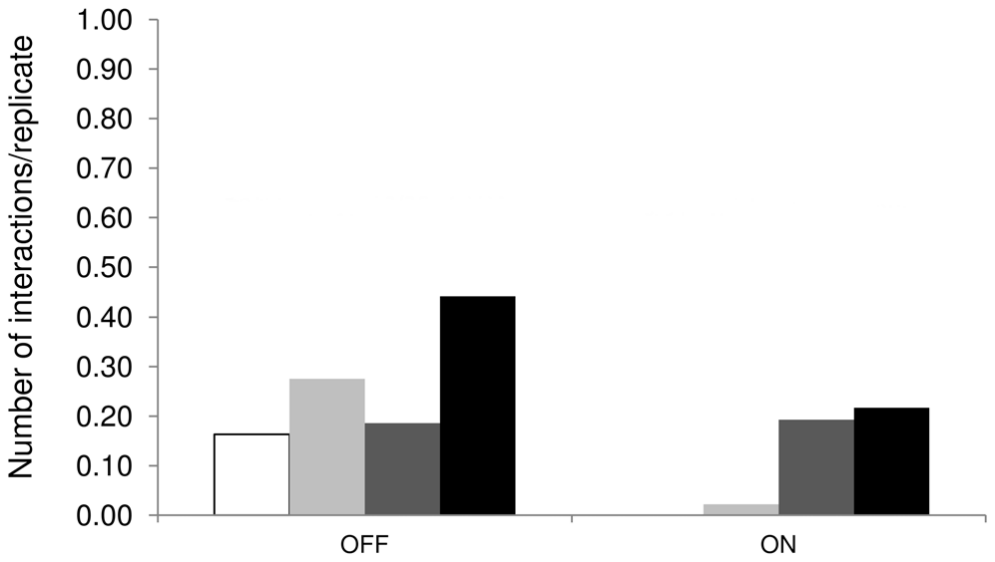
Proportion of breaches/tow (white), surface interactions/tow (light grey), underwater interactions/video (dark grey), and total number of interactions recorded (surface and on video)/video (black) when the electric field source was turned off or on. Reproduced and modified with permission from [52].

Ninety-eight of the 189 tows were performed with the EFS turned off and 91 with the EFS turned on. Due to logistical difficulties including electrodes wrapping around the equipment, poor visibility, and lack of light penetrating through the water surface, video footage was obtained from 169 tows. Eighty-six videos were taken with the EFS turned off and 83 with the EFS turned on during which 61 interactions (43 with EFS on, 18 with EFS off) between a shark and the decoy were recorded. Interactions visible from the surface accounted for 29 of the 61 interactions observed. The number of interactions per tow across all experiments was 0.32 and decreased from 0.44 to 0.20 when the electric field was produced. The strongest effects of the electric field were recorded for breaches, with no breaches observed when the electric field was produced compared with 16 breaches when the EFS was off. The number of surface interactions per tow decreased from 0.28 to 0.02 when the electric field was produced (Fig. 6; Table 4).

**Table 4:** Summary of the number of tows and interactions obtained when testing the deterrent on a dynamic decoy in South Africa.

	OFF	ON	Total
N^o^ of tows	98	91	189
N^o^ of videos	86	83	169
N^o^ of breaches	16	0	16
N^o^ of surface interaction	27	2	29
N^o^ of total interactions	43	18	61
N^o^ of breach/tow	0.16	0.00	0.08
N^o^ of surface interaction/tow	0.28	0.02	0.15
N^o^ of interaction on video	38	18	56
N^o^ of interaction on video/video	0.44	0.22	0.33

N^o^ of breach is the number of interactions during which a shark leaps out of the water, with several subtypes described by Martin et al. [44]; N^o^ of surface interaction is the number of interactions during which a shark does not leap out of the water but during which dorsal and/or caudal fins are visible above the water surface; N^o^ of underwater interaction is the number of interactions not visible from the surface.

The number of breaches per tow, surface interactions per tow, and total number of interactions recorded were significantly less with the electric field being emitted compared to when the EFS was turned off (Poisson exact test: *p*<0.001 for each test). The number of underwater interactions per video, however, did not change significantly whether the EFS was turned on or off (Poisson exact test: *p* = 1.00). Based on the probability of occurrence estimated when the EFS was off (0.16 and 0.28 for breaching and surface interaction, respectively), the probability of no breach, or two or less surface interactions occurring with the activated EFS was <0.001. It is therefore unlikely that the lack of breaches and small number of surface interactions observed with the electric field was due to chance alone.

Forty-seven of the 56 interactions observed on the underwater footage were considered assessable. Additional filtering following coding of the data resulted in 15% of the coding for behavioural approach (7 interactions) and 38% of the coding for change of direction (18 interactions) being removed. There was no difference in the amount of data filtered relative to the operational status of the EFS.

The proportions of aborted breaches increased when the EFS was turned on compared to when it was turned off (from 0.03 to 0.07). This was not significantly different (Poisson exact test: *p* = 0.54) due to the small number of aborted breaches coded (one each when turned on or off). The proportion of interactions where a sudden change of direction (used as a proxy for a reaction to the electric field) was not observed decreased from 0.59 to 0.27, but was not significantly different (Poisson exact test: *p* = 0.18) when the electric field was produced. The proportion of interactions where a sudden change of direction was observed increased significantly from 0.0 to 0.2 (Poisson exact test: *p* = 0.03). The proportion of interactions where the coder was ‘unsure’ if a sudden change of direction took place also increased from 0.03 to 0.13, but was not significantly different (Poisson exact test: *p* = 0.24).

## Discussion

Our study assessed the behavioural effects of the electric field produced by the Shark Shield Freedom7™. The study was performed in two locations and tested two distinct approach and behavioural situations to assess whether the response to the Shark Shield™ was consistent across behaviours. The electric field did not affect the proportion of static baits consumed, but significantly decreased the number of breaches, and surface interactions on a towed seal decoy. While the differences observed could be due to location or the different white shark populations [46], it is more likely related to the behavioural states being tested and associated energetic costs. Since rapid swimming is necessary to leave the water, the energy required for a breach is higher than that expended during inquisitive behaviour [47]. Considering the energetic cost of breaching, white sharks might be less likely to breach if they can sense any factor that could reduce their chance of being successful or which appears different to natural situations. On the other hand, a white shark might still be inquisitive around a static bait, regardless of the electric field because such approach requires similar energy expenditure to normal swimming. The inquisitive nature of the shark during the static bait trials is supported by the number of times the same white sharks were observed attempting to consume the bait (e.g., one white shark approached the static bait in 27 different trials, and another individual had 18 interactions in one trial).

The proportion of underwater interactions was expected to increase when the EFS was activated as a result of sharks aborting their predatory behaviour. The number of breaches and surface interactions decreased when the EFS was turned on, but it did not affect the number of underwater interactions. This suggests that white sharks either aborted their breaches outside of the range of the camera or did not initiate a breaching approach. On most days, the visibility was estimated to be less than 5 m indicating that sharks would have to be affected by the electric field further away than 5 m. This contradicts the results obtained from the static bait experiments, which indicate that the electric field did not affect white sharks further than a distance of two metres. The electric field might not reduce the consumption of bait two metres away from the EFS, but white sharks might detect the electric pulse from further away and prior to initiating the predatory attack. They might decide not to initiate a breaching approach, which would explain the reduction in breaches and surface interactions and the lack of associated increased number of underwater interactions.

White sharks may have become acclimatised to the electric field because of habituation to the electric field, or conditioning to the positive rewards resulting from consumption of the bait. For example, a shark that took the bait within the electric field may be more likely to take subsequent baits, because the discomfort caused by the electric field may not have been strong enough to counteract the reward. Such temporal correlation and decrease in the effectiveness of an electro-magnetic field has previously been observed in several species [48], [49]. This potential bias was examined but there was no strong decrease with time in the number of approaches per trial, interactions per approach, minimal distance, time to first appear, or time to take the bait. The lack of temporal correlations has also been observed in other species [22], [50]. It is likely that the small number of food rewards provided and the alternation of positive and negative reinforcements from the EFS being randomly activated for each trial prevented habituation from occurring and inducing any temporal effects in the study. The proportion of unidentified sharks (30.6%) may have impacted our ability to detect a decreasing response of individual sharks to the pulses. The issue of habituation or conditioning might have also occurred with the towed seal decoy. However, given the low number of interactions recorded when the electric field was present, the likelihood of habituation is low.

Experiments in South Australia may have been biased by interactions between sharks or the berley and bait used to attract white sharks, which may have modified the behaviour of the sharks on which the EFS was tested. Since it was not possible to know the location of all sharks present at the study site during the experiments, the impact of interactions between sharks could not be accounted for. However, while several sharks were observed within 20 m of the equipment, multiple sharks did not actively approach the bait simultaneously. The need for sufficient replicates to allow robust statistical analyses necessitates the use of berley to attract white sharks into the proximity of the EFS and observe their behavioural response. Regardless of the type of attractant used, our study shows that sharks are physically capable of being in close proximity (<0.5 m) to an EFS emitting a pulsed direct electric currents of ∼105 Vm^−1^ and of consuming baits *∼*2 m from the EFS.

The only previous study testing *in situ* behavioural responses of white shark to an electric field found an 80% reduction in the probability of a shark taking the bait [29]. This result contrasts with the results from the current study, which did not find any differences in the proportion of baits consumed. Because the electric pulse and waveform produced during the previous and present study were the same (Shark Shield Pty Ltd, pers. comm.), the disparity between these results is likely due the different configuration of the electrodes and position of the bait. Smit and Peddemors [29] attached the bait between the electrodes producing the electric field, whereas this study placed the EFS ∼230 cm away from the bait, similar to the way a diver would wear the product tested. Further testing should assess the impact that distance between the EFS and a bait has on the probability of the bait being consumed. This should be investigated against an accurate map of the electric field produced by the EFS to estimate the field strength at which white sharks first detect the field and at which they display a retreat response. This is yet to be carried out *in situ*, but studies in laboratory conditions have measured the minimum electric field strength that elicits a behavioural response for several shark species [13], [22], [23], [32]. The mean maximum field strength tolerated by hammerhead and leopard sharks before they displayed a retreat response was 18.50±13.27 and 9.64±10.28 Vm^–1^, respectively [32]. Both are higher than the threshold of 3–7 Vm^–1^ suggested by Smith [27] who investigated the use of an electric field to produce an electric barrier, supporting the idea that behavioural responses to electric fields varies between species [22], and that findings for one species should not be generalised to others.

Sharks were still capable of taking baits ∼230 cm away from the EFS, but the number of interactions within two metres of the EFS decreased when it was activated. Such a reduction in the number of interactions towards a stimulus placed two metres away from the EFS has previously been observed in other species (e.g., Galapagos sharks (*Carcharhinus galapagensis*) (Robbins, unpublished data). Although behavioural effects two metres from the EFS were observed in both studies, white sharks were observed less than 0.5 m from the EFS on several occasions (e.g., Video S10), and Galapagos sharks consumed sardines (*Sardinops sagax*) two metres away from an EFS (Robbins, unpublished data). Scalloped hammerhead sharks (*Sphyrna lewini*) and leopard sharks (*Triakis semifasciata*) were affected by a strong pulsed electric field, but were also able to swim through the electric field and into voltage gradients greater than 30 V/m [32]. Four species of small benthic rays and sharks, fiddler ray (*Trygonorrhina fasciata*), eagle ray (*Myliobatis australis*), yellowback stingaree (*Urolophus sufflavus*), and spotted catshark (*Asymbolus* rubiginosus), have been observed to approach a bait positioned next to the same electric field as used in the present study [51]. These studies confirm that electric fields can affect the behaviour of sharks, but that some rays and sharks, including white sharks, are able to be in close proximity to the EFS and consume baits close to electric fields.

During this study, white sharks took twice as long to take the bait when the EFS was activated compared to when it was not. An increase in the time it takes to consume a bait is consistent with findings for Galapagos sharks (Robbins, unpublished data). The number of interactions per approach also increased when the EFS was activated, similar to the previous study on white sharks [29]. This suggests that even though white sharks are able to consume baits close to a strong electric field, it can affect white shark behaviour and result in some sharks hesitating and taking longer to consume the bait.

The behavioural responses observed in the present study varied across individuals, with some sharks less affected by the electric field than others. The reason for this variation is unknown and may be a combination of motivation, different natural feeding histories, dominance hierarchies, individual experiences, or behavioural syndrome (consistency of responses across situations). Intra-specific variability was also noted for hammerhead and leopard sharks, as seen by the large standard deviations of the maximum voltage gradient and the difference in the voltage gradient required to elicit head twitches [32]. The electric deterrent tested produced a behavioural reaction in some sharks, but cannot be relied on to prevent shark attacks in all situations.

This study indicates that the behavioural response of white sharks and the level of risk reduction resulting from the electric field is contextually specific, and depends on the motivational state of sharks. The electric field we tested had an effect on white shark behaviour up to two metres from the EFS and reduced the incidence of predatory strike, but did not deter or repel this species in all situations nor did it repel all individuals. Given that the static bait experiments showed that the electric field did not reduce the likelihood of baits being taken, the effects observed in the seal decoy study are likely to be situation-specific. The large discrepancy in the findings from the present study compared to those of Smit and Peddemors [29] also highlights the need for future studies to focus on testing the effects of electric fields at different distances from the EFS. An accurate map of the electric field produced by different voltage strengths would also aid in determining the electric current levels eliciting behavioural response and the distance from which white sharks can be expected to first detect and react to the electric field. Finally, the study was undertaken on white sharks and further study should include other elasmobranch species, as the behavioural responses to electric fields are known to vary across species [22].

## Supporting Information

Video S1 **Example of a white shark taking the bait preceded by one approach and one interaction.**
(AVI)

Video S2 **Example of a white shark taking the bait preceded by one approach and six interactions.**
(AVI)

Video S3 **Example of interaction categorised as a breach.**
(MPG)

Video S4 **Example of interaction categorised as a breach.**
(AVI)

Video S5 **Example of interaction categorised as a surface interaction.**
(AVI)

Video S6 **Example of interaction categorised as an underwater interaction.**
(AVI)

Video S7 **Example of interaction categorised as an investigation.**
(MPG)

Video S8 **Example of interaction categorised as an aborted breach.**
(MPG)

Video S9 **Example of interaction with a change of direction (proxy for a reaction to the electric deterrent).**
(WMV)

Video S10 **Example of interaction during which a shark approached within 0.5 m of an activated electric deterrent.**
(AVI)
